# Stages of Behavior Change for Physical Activity in Airport Staff: a quasi-experimental study

**DOI:** 10.17533/udea.iee.v38n1e02

**Published:** 2020-02-26

**Authors:** Khalil Mahmoudi, Ali Taghipoor, Hadi Tehrani, Hadi Zomorodi Niat, Mohammad Vahedian-Shahroodi

**Affiliations:** 1 M.Sc. Student. Department of Health Education and Health Promotion, Mashhad University of Medical Sciences, Mashhad, Iran. Email: MahmoudiKH1@mums.ac.ir Mashhad University of Medical Sciences Mashhad University of Medical Sciences Mashhad Iran MahmoudiKH1@mums.ac.ir; 2 Associate Professor, Epidemiology, Social Determinants of Health Research Center, Mashhad University of Medical Sciences, Mashhad, Iran. Email: taghipoura@mums.ac.ir Mashhad University of Medical Sciences Mashhad University of Medical Sciences Mashhad Iran taghipoura@mums.ac.ir; 3 Assistant professor of Health Education and Health Promotion, Social Determinants of Health Research Center, Mashhad University of Medical Sciences, Mashhad, Iran. Email: Tehranih@mums.ac.ir Mashhad University of Medical Sciences Mashhad University of Medical Sciences Mashhad Iran Tehranih@mums.ac.ir; 4 M.Sc. Department of Management, School of Health, Mashhad University of Medical Sciences, Mashhad, Iran. Email: ZomorrodiNH1@mums.ac.ir Mashhad University of Medical Sciences Mashhad University of Medical Sciences Mashhad Iran ZomorrodiNH1@mums.ac.ir; 5 Associated Professor of Health Education and Health Promotion, Social Determinants of Health Research Center, Mashhad University of Medical Sciences, Mashhad, Iran. (Corresponding author) Email: Vahedianm@mums.ac.ir Mashhad University of Medical Sciences Mashhad University of Medical Sciences Mashhad Iran Vahedianm@mums.ac.ir

**Keywords:** health education, exercise, airports, control groups, surveys and questionnaires, models, theoretical., educación en salud, ejercicio, aeropuertos, grupos control, encuestas y cuestionarios, modelos teóricos., educação em saúde, exercício, aeroporto, grupos controle, inquéritos e questionários, modelos teóricos.

## Abstract

**Objective.:**

This work sought to determine the effect of an educational intervention based on the stages of change in promoting physical activity in employees in the Mashhad airport in Iran.

**Methods.:**

This was a quasi-experimental study conducted with the participation of 60 volunteers (30 in the intervention group and 30 in the control group) who were in the stages of contemplating or preparing for change in physical activity. The intervention consisted in educational activities provided during home visits, telephone calls, group training sessions, and delivery of printed material. To gather the information, the study used five questions on the stage in which they were for behavioral change in physical activity, according to the Theoretical Model by Marcus *et al.,* (1. pre-contemplation, 2. contemplation, 3. preparation, 4. action, and 5. maintenance), and the International Questionnaire on Physical Activity. Changes in the stages were evaluated during three moments: upon entering the study, at the end of the intervention (8^th^ month), and two months after the second evaluation (10^th^ month).

**Results.:**

During the 10th month evaluation, it was noted that 26.7% of the subjects from the intervention group versus 3.3% from the control group improved their physical activity and were in the action stage (*p*<0.01).

**Conclusion.:**

The educational intervention based on stages of change is effective in promoting physical activity in the participants and may be used in educational programs that seek to improve physical activity in the employees studied.

## Resumen

**Objetivo.:**

Determinar el efecto de una intervención educativa basada en las etapas de cambio en la promoción de la actividad física en los empleados del aeropuerto de Mashhad en Irán.

**Métodos.:**

Estudio cuasiexperimental realizado con la participación de 60 voluntarios (30 en el grupo de intervención y 30 en el grupo control) quienes se encontraban en las etapas de contemplación o preparación para el cambio en la actividad física. La intervención consistió en actividades educativas proporcionadas durante visitas domiciliarias, llamadas telefónicas, sesiones de capacitación en grupo y entrega de material impreso. Para la recolección de información se utilizaron 5 preguntas sobre la etapa en la que se encontraba para el cambio de comportamiento en la actividad física, según el Modelo Transteórico de Marcus *et al.* (1. pre-contemplación, 2. contemplación, 3. preparación, 4. acción y 5. mantenimiento), y el Cuestionario Internacional de Actividad Física. Los cambios en las etapas se evaluaron en 3 momentos: al ingreso al estudio, al finalizar la intervención (mes 8) y dos meses después de la segunda evaluación (mes 10).

**Resultados.:**

En la evaluación de los 10 meses se apreció que el 26.7% de los sujetos del grupo de intervención versus el 3.3% del grupo control mejoraron su actividad física y se encontraban en la etapa de acción (*p*<0.01).

**Conclusión.:**

La intervención educativa basada en las etapas de cambio es efectiva en la promoción de la actividad física en los participantes por lo que puede ser empleada en programas educativos que tengan como objetivo mejorar la actividad física de los empleados

## Resumo

**Objetivo.:**

Determinar o efeito de uma intervenção educacional baseada nos estágios de mudança na promoção da atividade física nos funcionários do aeroporto de Mashhad, no Irã.

**Métodos.:**

Estudo quase experimental realizado com a participação de 60 voluntários (30 no grupo intervenção e 30 no grupo controle) que estavam nos estágios de contemplação ou preparação para a mudança na atividade física. A intervenção consistiu em atividades educativas realizadas durante visitas domiciliares, telefonemas, sessões de treinamento em grupo e entrega de material impresso. Para a coleta de informações, foram utilizadas 5 questões no estágio em que se destinava à mudança de comportamento na atividade física, conforme o Modelo Transteórico de Marcus et al. (1. pré-contemplação, 2. Contemplação, 3. Preparação, 4. Ação e 5. Manutenção) e o Questionário Internacional de Atividade Física. As alterações nas etapas foram avaliadas em três momentos: na admissão ao estudo, ao final da intervenção (mês 8) e dois meses após a segunda avaliação (mês 10).

**Resultados.:**

Na avaliação de 10 meses, observou-se que 26.7% dos indivíduos no grupo intervenção versus 3.3% no grupo controle melhoraram a atividade física e estavam no estágio de ação (*p*<0.01).

**Conclusão.:**

A intervenção educacional baseada nos estágios de mudança é eficaz na promoção da atividade física dos participantes e pode ser usada em programas educacionais que visam melhorar a atividade física dos funcionários.

## Introduction

Due to the increasing mechanical life and the rapid development of today's world, physical activity has been considered important and lack of attention to this important cause increased several diseases,(1) especially in employees, like airport staff who may sit for continuous long hours and may be exposed to high stress. Sedentary lifestyle has been known as risk factor for various diseases. In many areas of health care in health care programs, the physical and psychological benefits of regular physical activity in reducing mortality has been proven with adequate reasons.(2) Physical inactivity or lack of physical activity is the fourth risk factor for mortality in the world, which includes 6% of mortality worldwide and approximately 3.2 million deaths occur each year because of it. In 2016, 71% of worldwide deaths was from non-communicable diseases (NCD).(3) According to the World Health Organization, in 2020 the rate is estimated to be at 73% (three quarters of all deaths) and 60% of the (burden of diseases), respectively.(4)

The findings of the national health survey among Iranian adults show that >80 percent of the Iranian population is physically inactive and has inactivity lifestyles so that 44.4% of Iranians never practice sports during their leisure time.(5) Given that today many jobs are sedentary (sitting), employees are also at risk of illness due to inactivity. Physical activity not only improves the performance and health of employees, but also increases production, reduces injury, creates spirit of cooperation, and increases communication and job satisfaction.(6) 

One of the models used in health education and health promotion is the stage of change model or trans-theoretical model introduced in the late 1970s by Prochaska. A trans-theoretical model is a model sensitive to minor variations in the progress of a behavior; it is much more practical to measure physical activity compared to other patterns of behavioral change that are viewed by all or not.(7) Among the important structures of the model is stage of change, which to understand and predict health behaviors focuses on the cognitive factors affecting the decision that people protect themselves from traumatic events. The stage of change suggests time dimension and means that change occurs over time.

The stage of change suggests that the person is not ready to change, or the person is not placed in at least the same level of readiness, so people should be intervened as different from one another according to the stage of their change; these stages are the conditions of motivation including five steps: pre-contemplation, contemplation, preparation, action, and maintenance. Within this structure, the pre-contemplation stage is defined as the stage where the person has still not thought about changing or adopting a behavior at least for the next six months.(8) In the contemplation stage, the person really thinks of changing the behavior during the next six months, but is not yet prepared to take the necessary action. In the preparation stage, people seriously think about changing behaviors and want to make changes in the near future (normally in the following month). The action stage is the stage where the person has created appropriate changes in lifestyle during the past six months. In the maintenance stage, we see a longer period of strengthening the behavioral changes (>6 months), but active and conscious effort is needed to maintain it.(9) Prochaska believes this pattern has been successfully applied in health education interventions.(10)

Familiarization by nurses with the model of change stages is important because it considers behavioral change as a stage process; to change people's behaviors towards healthy behaviors, interventions proportional to the preparation stage of individuals and helping them to go through various stages are needed.(11,12) The aim of this study was to determine the impact of the effect of education based on the structure of stage of change in physical activity promotion of Mashhad Airport staff in Iran and it is expected that the results of this study could provide a suitable and applied solution to increase physical activity among airport employees.

## Methods

This was a quasi-experimental interventional study with case group. The researched community was employees from the Mashhad Airport staff in Iran, during 2018. In determining the sample size, to achieve the average effect size of 0.6 for each group(13) at least 40 subjects were allocated to each group. Data collection from the airport staff was done randomly, so after an initial review and data analysis based on stage of change, among airport employees who were in the contemplation and preparation groups, 30 were selected as the intervention group and 30 were selected as the control group.

The intervention and control groups were selected randomly; hence, the two groups were not related to each other and were in different work shifts. The control group was chosen in such a way that they did not have a common place of work with the intervention group or in a shift that did not belong to the intervention group in that shift. On the first session (30 min), the participants were informed of the importance of the study and the objectives and how to answer the questionnaire’s questions. The intervention program was conducted with the intervention group. To evaluate the effectiveness of the intervention immediately and two months after the intervention, the data were re-collected with the same questionnaire. Descriptive and analytical tests, such as Chi-square, paired t-test, and independent t-test were used to determine the difference among the distribution of variables between the intervention and control groups and data were analyzed via SPSS software version 16. Inclusion criteria involved having at least one year of work experience, having a mobile phone, and having no restrictions or prohibitions on engaging in physical activity, which were verified through interview and informed consent to participate in the research. Exclusion criteria involved the reluctance to cooperate with the researcher and failure to answer at least 10% of the questions. 

Data were collected through a researcher-made questionnaire including demographic data, stage of change questions, and changes to the native version of the International Physical Activity Inventory,(14) which included the following sections: a) demographic information containing questions in terms of age, level of education of subject and wife, marital status, monthly income; b) based on stage of change in physical activity in trans-theoretical model consisted of five questions measured by a five-item scale (yes or no) prepared by Marcus *et al*.,(14) that the stages based on physical activity included: 1. pre-contemplation stage, 2. contemplation stage, 3. preparation stage, 4. action stage, and 5. maintenance stage; c) short form of the International Physical Activity Questionnaire (IPAQ), which includes seven questions about high and moderate physical activity, sitting, and walking in the last seven days. Intensity of physical activity for each activity is calculated by metabolic equivalent minutes/week. Self-reported data from physical activity are collected by the short form of the standard physical activity questionnaire. The international physical activity questionnaire was expanded by a group of experts in 1998 seeking to show the importance of physical activity to facilitate the study of physical activity based on international standards.(15) This questionnaire has been validated by Karimzadeh in Iran.(16) The original authors recommended the short version of the physical activity questionnaire for researches in physical activity because this questionnaire takes shorter time compared to the long form and answers the questions more accurately and more completely. The international physical activity questionnaire is translated into different languages, including Persian.(17)

### Description of the Educational Intervention.

For the intervention group, the educational content was provided by the researcher during three 30-min training sessions per week. For this purpose, the educational content was compiled as a CD-ROM and autographed according to the headings of the Ministry of Health and Medical Education on physical activity and was provided to the intervention group on a daily basis and monitored weekly by short message services (SMS) for four times. The activities involving the study groups are detailed in Table 1.

**Table 1:** Activities involving the study groups

Stages	Groups	Groups	Description
Stages	Intervention	Control	Description
1	Enrollment	Enrollment	During the first month of the research, the informed consent is signed and sociodemographic and clinical data are collected.
2	Measuring of self-care behaviors	Measuring of self-care behaviors	For all patients, baseline self-care behaviors were measured upon enrollment. In the intervention group, the second measurement happened before the start of the second educational meeting (8^th^ month) and, in the control group, before the only meeting (9^th^ month).
3	Home visit	No	Home visits take place in months 1 and 8, during which the patient’s basic social conditions for health care are evaluated. The family and patient receive indications from the nurse to improve self-care.
4	Telenursing	No	In months 2, 3, 4, 5, 6, and 7 self-care was evaluated by phone, using a guide to monitor the nursing plan recommended during the previous contact.
5	Educational meeting at the start of the research	No	This took place during the first month. Patients and their families share experiences and knowledge about what heart failure is, care for the disease, importance of physical exercise, and stress management techniques. A workshop on healthy cooking is offered afterwards.
6	Educational meeting at the end of the research	Educational meeting at the end of the research	This took place in month 8 in the intervention group, returning to the self-care behavior aspects observed during telenursing that caused most difficulties; before the educative activity, self-care behaviors are measured. In the control group, this activity took place in month 9, involving the same activities as during the first educational meeting with intervention group patients.
7	Distribution of educational brochure during the first educational meeting	Distribution of educational brochure at the end of the research	The brochure didactically describes how to plan activities to avoid fatigue, general aspects like diet, and alarm signs of Heart Failure Decompensation, when to ask for help, adaptation to the therapeutic regime, weight control and ingested and eliminated fluid. In addition, the brochure contains a contract for patients to sign to take care of themselves. This brochure also contains tables to control weight, ingested and eliminated fluids, and medication administration.

This article is part of the results of a dissertation approved by the Master’s degree in Health Education and Health Promotion of Mashhad University of Medical Sciences, approved by the Ethics Committee of the Faculty of Medicine of the university with IR.MUMS.REC.1395.236. Ethical considerations of this research include providing a written letter of permission and obtaining permission from the Mashhad Airport's General Office to conduct the research, introducing itself to each of the research units and explaining the objectives and nature of the research, assuring research units regarding the confidentiality of information, satisfaction, respect for trust and honesty in reviewing texts and analyzing information.

## Results

In this study, 60 airport employees were entered and were divided into two groups. These two groups had the same demographic variables and were followed up till the end of the study.

Table 2 shows that no statistically significant difference existed in the general characteristics of the study groups.

**Diagram 1:**
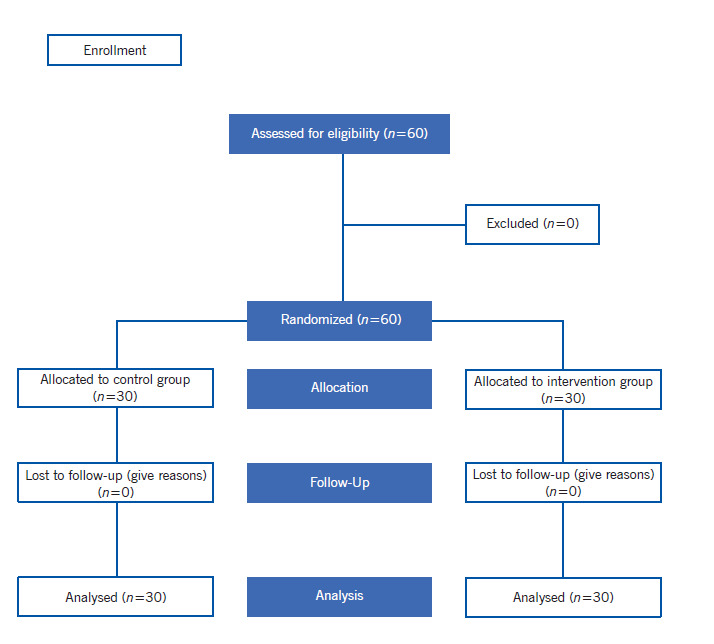
Sample Selection Stages and Follow-ups

The characteristics of the whole group prevailed with age between 31 and 40 years (61.4%, mean of 33 ± 2.7 years), married marital status (70.2%), with one to two children (56.1%), official or A treaty employment (31.6% each), with work experience between 6 and 10 years (36.8%), and Bachelor’s degree (93%).

The mean age of airport employees was 34.7 ± 12.8 years old and 92% were male and the rest were female. Most of the employees (57%) had undergraduate and graduate degrees, and the majority were married (87%). The results showed that both groups had no significant difference in terms of demographic variables and none of the participants left the study (Table 2). 

**Table 2:** Frequency distribution of demographic information in the intervention and control groups

Variable	Control *n*=30	Intervention *n*=30	***p* value**
	***n* (%)**	***n* (%)**	
Age (years)			0.35
Less than 35	19 (63.3)	15 (50)	
35 to 45	8 (26.7)	8 (26.7)	
More than 45 years	3 (10)	7 (23.3)	
Marital status			0.67
Married	28 (93.3)	26 (86.6)	
Single	2 (6.7)	4 (13.4)	
Gender			0.69
Male	28 (93.3)	28 (93.3)	
Female	2 (6.7)	2 (6.7)	
Education grade			0.67
Under the Diploma	4 (13.3)	2 (6.7)	
Diploma	9 (30)	7 (23.3)	
Undergraduate and Bachelor	15 (50)	17 (56.6)	
More than Bachelor	2 (6.7)	14 (13.4)	
Service record			0.14
Less than 10 years	17 (56.7)	13 (43.4)	
Between 10 and 20 years	10 (33.3)	8 (26.60	
More than 20 years	3 (10)	9 (30)	
Spouse education			
Under the diploma	4 (13.4)	2 (6.6)	0.08
Diploma	10 (33.3)	6 (20)	
Undergraduate and Bachelor	13 (43.3)	15 (50)	
More than Bachelor	1 (3.3)	3 (10)	

According to the findings in Table 3, the subjects of the intervention and control groups were selected with respect to stage of change from the contemplation and preparation groups. Immediately, and after two months of intervention, 29 people (97%) of the subjects in the intervention group changed level and entered the higher stage in terms of the level of physical activity in stages of change. After the intervention, only one person (3%) of the control group changed level and entered the higher stage, and others remained at the previous level. Independent t-test results showed that before the intervention, two groups were homogeneous in terms of stage of change. The results of chi-square test showed significant differences in frequency changes in the stages of change on the intervention group immediately and two months after the intervention.

**Table 3:** Comparison of the frequency of stage of change before, immediately after, and two months after the intervention between both groups

Stage of change	Intervention	Intervention	Intervention	Control	Control	Control
Stage of change	Before the intervention *n* (%)	Immediately after the intervention *n* (%)	Two months after the intervention *n* (%)	Before the intervention *n* (%)	Immediately after the intervention *n* (%)	Two months after the intervention *n* (%)
Contemplation	13 (43.3)	1 (3.3)	1 (3.3)	11 (36.7)	9 (30)	10 (33.3)
Preparation	17 (56.7)	24 (80)	21 (70)	19 (63.3)	21 (70)	19 (63.3)
Action	0 (0)	5 (16.7)	8 (26.7)	0 (0)	0 (0)	1 (3.3)
Test result	X^2^=24.16 *p*<0.001	X^2^=24.16 *p*<0.001	X^2^=24.16 *p*<0.001	X^2^=0.89 *p*=0.64	X^2^=0.89 *p*=0.64	X^2^=0.89 *p*=0.64

## Discussion

This study investigated the effect of education based on stage of change in physical activity promotion of Airport staff and the results indicated the effectiveness of principal counseling in this regard. In the trans-theoretical model, the stage of change is the strongest predictor of physical activity.(18) Therefore, to promote physical activity in people, this structure should be especially considered in educational interventions.(19) Interventions performed based on the structure on stage of change have more effectiveness in facilitating behavioral change compared to other studies. In other words, interventions focusing on stage of change and having a specific training program for each step should have more positive outcomes.(20)

In this study, based on stages of change in physical activity, the educational program was performed for individuals in the contemplation and preparation stages. After performing the educational program, significant difference was observed in the stages of change in the intervention group; this difference was not significant in the control group that reflects the impact of the intervention. In the intervention group, 40% of individuals in the contemplation stage were reduced and moved to higher stage of engaging in regular physical activity. In the interventions based on stage of change, the main purpose is that of reducing the number of people in the inactive stages and increasing the number of people in the preparation and action stages. These results are similar in many interventional studies conducted in this area.(20-22) 

In terms of the transition from the contemplation stage to the action stage, Tehrani *et al.,*(21) showed that the educational intervention was effective on the process of transition from pre-contemplation and contemplation stages to the action stage, so that 83% of the intervention group and 17% of the control group had entered the action and maintenance stages after one year of follow-up.

There was no significant difference in the physical activity of both groups before the intervention, but after the intervention, the mean of the physical activity score in the intervention group was significantly higher than in the control group, indicating the effect of the educational program on increasing physical activity. The study by Tehrani *et al.* (21) confirmed our findings, so that their results showed that the amount of physical activity before the intervention was not significantly different in the intervention and control groups, but the independent t-test results showed that the mean score of physical activity after the intervention had significant difference between both groups, and the mean of the above mentioned significantly increased in the intervention group. Also, the findings by Vafaee *et al.,* (22) confirmed the findings of the present study, showing that comparing the groups indicated that a greater proportion of the cases in the intervention group (75%) were in middle level of physical activity, which represents an increase in mean physical activity after the intervention in the intervention group. In the study by Mardani,(23) physical activity increased after the intervention, consistent with the present study.

This study confirms the effectiveness of systematic educational programs based on stages of change to promote physical activity in Mashhad airport staff. It seems that stages of change can be used as a framework to design educational programs to improve employees' physical activity and reduce sedentary lifestyle-related diseases.

Considering the effectiveness of the educational intervention to increase the physical activity of the staff, educational programs for all employees in the airport should be encouraged. 

The limitations of this study were to collecting the information on the amount of physical activity through self-reporting, which tried to minimize the participants by trusting the participants. It is suggested that interview method be used in future researches and the results compare with self-reporting method.
